# Child Head Circumference and Placental MFSD2a Expression Are Associated to the Level of MFSD2a in Maternal Blood During Pregnancy

**DOI:** 10.3389/fendo.2020.00038

**Published:** 2020-02-05

**Authors:** María Sánchez-Campillo, María Ruiz-Palacios, Antonio J. Ruiz-Alcaraz, María Teresa Prieto-Sánchez, José Eliseo Blanco-Carnero, Matilde Zornoza, María José Ruiz-Pastor, Hans Demmelmair, Manuel Sánchez-Solís, Berthold Koletzko, Elvira Larqué

**Affiliations:** ^1^Department of Physiology, Faculty of Biology, Regional Campus of International Excellence Campus Mare Nostrum, University of Murcia, Murcia, Spain; ^2^Department of Biochemistry, Molecular Biology B and Immunology, Faculty of Medicine, Regional Campus of International Excellence Campus Mare Nostrum and Biomedical Research Institute of Murcia (IMIB), University of Murcia, Murcia, Spain; ^3^Hospital Clínico Universitario Virgen de la Arrixaca, Murcia, Spain; ^4^Ludwig-Maximilians-University Munich, Dr. von Hauner Children's Hospital, München, Germany

**Keywords:** pregnancy, gestational diabetes mellitus, neurodevelopment, biomarkers, MFSD2a, fatty acids transport, docosahexaenoic acid

## Abstract

Gestational diabetes mellitus (GDM) is a world-wide health challenge, which prevalence is expected to increase in parallel to the epidemic of obesity. Children born from GDM mothers have lower levels of docosahexaenoic acid (DHA) in cord blood, which might influence their neurodevelopment. Recently, the membrane transporter Major Family Super Domain 2a (MFSD2a) was associated with the selective transportation of DHA as lysophospholipids. The expression of the DHA membrane transporter MFSD2a is lower in GDM placentas, which could affect materno-fetal DHA transport. Humans with homozygous inactivating mutations in the MFSD2a gene present severe microcephaly and intellectual impairments. Herein, we intended to identify early blood biomarkers that may be of use during pregnancy to monitor the offspring development and the adequate nutritional interventions, such as nutritional supplementation, that may be selected to improve it. We evaluated MFSD2a expression in maternal blood at the third trimester of pregnancy, and its potential relationship with the expression of placental MFSD2a at delivery and child outcomes. Three groups of pregnant women were recruited: 25 controls, 23 GDM with dietary treatment, and 20 GDM with insulin treatment. Maternal and neonatal anthropometric and biochemical parameters were evaluated. MFSD2a was analyzed in placenta, blood and serum. MFSD2a protein expression in maternal blood was significantly lower in GDM groups and correlated with placental MFSD2a and Z-score neonatal head circumference during the first 6 months of life. The cord/maternal serum ratio of DHA, a solid indicator of materno-fetal DHA transport, was reduced in GDM groups and correlated with MFSD2a in maternal blood at the third trimester and in placenta at delivery. This indicates that altered MFSD2a levels in maternal blood during pregnancy might influence placental nutrient transport and fetal neurodevelopment. Furthermore, MFSD2a levels in maternal blood on the third trimester were inversely correlated to DHA in maternal serum lyso-PL. Thus, the level of MFSD2a in maternal blood could be used as a potential biomarker for the early detection of disturbances of MFSD2a expression during pregnancy and the subsequent consequences for the neurodevelopment of the child, as well as it may help to choose the optimal treatment approach for the affected subjects.

## Introduction

Gestational Diabetes Mellitus (GDM) is a world-wide health challenge, and its occurrence is estimated to further increase together with the epidemic of obesity (1). The treatment of GDM is initially based on diet modification and increased physical activity, and insulin therapy when necessary (2). GDM may produce several effects in the offspring, as it enhances the risk of macrosomia in children, shoulder dystocia, neonatal hypoglycaemia, hyperbilirubinemia, and even the development of type 2 diabetes mellitus early in life (1, 3). Furthermore, several studies have reported potential adverse consequences for the neurodevelopment of GDM offspring at school age (4–6), but the reasons for this cognitive delay are not totally understood yet.

Long chain polyunsaturated fatty acids (LC-PUFA), and specially docosahexaenoic acid (DHA, 22:6n-3), have important effects on membrane function and neurogenesis processes in early life (7). DHA acyl chains promote the function of the G-protein-coupled system in membranes of photoreceptor cells and enhance the signaling pathways of metarhodopsin II (8). The prenatal and postnatal accretion of LC-PUFA determines myelination and synaptogenesis during postnatal brain growth spurt (9). Several studies, but not all, reported improvements of the offspring in some neurodevelopment tests as a result of DHA supplementation during gestation, or, at least, positive relationships between maternal or cord serum DHA percentages and cognitive skills in young children (10).

By using *in vivo* studies with stable isotopes, we have previously demonstrated an impaired maternal-fetal transfer of DHA in women with GDM (11). Observational studies also confirm a reduction of DHA in cord blood of GDM (12, 13). Lower DHA levels in cord blood of GDM were directly associated to the psychomotor score from Bayley's test and intraday variability rhythm of activity in children at 6 months of age (14). These data confirm a key role of this fatty acid in the neurodevelopment of these babies.

Recently, the protein Major Facilitator Superfamily Domain containing 2A (MFSD2a) was characterized as a primary carrier for the uptake of DHA and other long-chain fatty acids as lyso-phospholipids (lyso-PL) into the brain (15) and the eye (16). MFSD2a is an orphan carrier that plays a dual role in brain, establishing integrity of the blood-brain barrier and the uptake of unsaturated lyso-PL as DHA (15, 17). MFSD2a knock-out mice show reduced levels of DHA in brain accompanied by neuronal cell loss in hippocampus and cerebellum, and exhibit severe microcephaly, as well as deficits in both learning and memory (15). Moreover, humans with homozygous inactivating mutations in the MFSD2a gene present severe microcephaly and intellectual impairments (18–20). Thus, it is of great interest to detect altered MFSD2a levels during pregnancy in key tissues obtained from non-invasive human samples such as the blood.

MFSD2a is also the orphan receptor of Syncytin-2, which is involved in the fusion of cytotrophoblats in the placenta (21). MFSD2a protein is expressed in the majority of organs and tissues, presenting high level of expression in placenta (22). The decrease of MFSD2a expression in GDM placentas has been previously described by our group using Western blotting analyses (23), and also by other authors who have studied both gene and protein expression levels (24). Moreover, mRNA and protein levels of MFSD2a were markedly lower in severe pre-eclampsia placenta but not in moderate pre-eclampsia (25). Pre-eclampsia involve lower DHA levels in cord blood. Thus, clinical biomarkers of these and other related malfunctions during pregnancy are of great interest.

The aim of this study was to evaluate whether MFSD2a levels in maternal blood during the third trimester of pregnancy could be also used to detect the appearance of disturbances in the expression of MFSD2a in placental tissue at delivery, and if it could be associated with fetal development. Thus, the measurement of maternal blood MFSDa levels could be used as a biomarker to detect early abnormal syncytiotrophoblast formation, and/or impaired nutrient transportation across the placenta, which would affect normal fetal neurodevelopment in the offspring of GDM patients.

## Materials and Methods

### Study Population

Eligible for the study were pregnant women who fulfilled the following inclusion criteria: singleton pregnancy, age 18–40 years, non-smoking, omnivorous diet, and fetal Doppler scan within normal reference range at the time of recruitment. The subjects were recruited in their third trimester of gestation (28–32 weeks) between years 2008 and 2010 in the Obstetrics and Gynecology Service of the “Hospital Clínico Universitario Virgen de la Arrixaca” in Murcia, Spain. The study protocol was approved by the Hospital Ethics Committee in accordance with the Declaration of Helsinki, and written informed consent was obtained from all the participating women after explanation of the study. Three groups of pregnant women were established: 25 healthy controls, 23 women with diagnosed gestational diabetes mellitus receiving dietary treatment (GDM-Diet), and 20 women with diagnosed gestational diabetes mellitus who required insulin treatment for their glycemic control (GDM-Insulin).

The control group was recruited from healthy pregnant women submitted to routine ultrasound in the weeks 20–22 of gestation. During the third trimester, we contacted those women who showed interest in participating in the study. GDM was diagnosed between weeks 24 and 28 of gestation by screening with an oral challenge of 50 g glucose (O'Sullivan test) (26). A positive screening result (1 h plasma glucose concentration > 140 mg/dL) was followed by a 3 h oral glucose tolerance test with 100 g of an oral glucose load and further plasma glucose analyses at 1, 2, and 3 h after ingestion. The test was considered positive if two out of the four serum glucose values were above the cut off (basal: 105 mg/dL, 1 h: 190 mg/dL, 2 h: 165 mg/dL, and 3 h: 145 mg/dL) according to the criteria of the National Diabetes Data Group (27). The recruitment of the GDM-insulin group started generally later than the GDM-Diet one since we had to test first whether subjects were able to achieve good glycemic control with the dietary intervention, but it took place always before week 32 of gestation. The treatment time ranged between 8 and 12 weeks.

Subjects in the GDM–Diet group did not require insulin treatment or antidiabetic agents until delivery. The choice of treatment allocation was determined by the attending physician and unrelated to the study. Initially, all patients received a treatment based on diet and exercise. The number of calories of the diet depended on their weight gain until that moment, and changes were done related to carbohydrate consumption. Moreover, a reflectometer was provided to the subjects in order to record their glucose levels two times per day, one preprandially and one postprandially. After 2 weeks, these data were revised by the physician, and those subjects who achieved good glycemic control were assigned to the GDM-Diet group, while those who did not achieved them were assigned to the GDM-Insulin group. A good glycemic control was considered when glucose levels did not exceeded a preprandial cut off 90 mg/dL and postprandial cut off 120 mg/dL more than two times a week. The insulin treatment consisted of low doses of rapid-acting insulin during meals (no more than 10 units). No antidiabetic drugs were used in any of the groups.

### Maternal and Child Anthropometrical Measurements

Maternal height, weight, waist, and hip circumferences, and blood pressure were measured at the time of delivery. Maternal age, parity, and pregestational weight were also recorded.

An ultrasound scan (Voluson 730 Pro, General Electric Medical Systems, Kretz Ultrasounds, USA) was used both at recruitment and at week 38 of gestation to obtain the fetal abdominal circumference (AC) and placental thickness. The fetal biometry Z-score was calculated using the tables of the Institute of Child Health of London (28).

Anthropometrical variables of the neonate (weight, length, body mass index or BMI, and head circumference) were measured at birth, and the Z-score was calculated using Spanish reference data (29). Follow-up checks of children, including a complete physical examination of the patient, and the assessment of the type of feeding and somatometry, were also performed at 15 days, and at 1, 3, 6, and 12 months.

### Sampling

Samples of 10 mL of maternal blood were collected both at recruitment (28–32 weeks) and during labor. Blood samples were collected under fasting conditions at recruitment, but not at delivery. At delivery, an additional sample of 2 mL of venous cord blood was also collected. An aliquot of 1 mL of maternal blood was directly stored at −80°C. From the rest of the blood, serum was separated within 1 h, by centrifugation at 120 × *g* for 5 min. An aliquot of at least 200 μL of serum was frozen subsequently at −80°C for later fatty acid analysis and Western blotting assays, while the rest was used for clinical biochemical analyses performed in the hospital.

The total placenta weight was recorded immediately after delivery. Samples of 1 × 1 × 1 cm of villous from placental central cotyledons were cut and stored at −80°C until later analysis.

### Biochemical Analysis

Serum glucose and triglycerides (TG) were measured using an automatic analyzer (Roche-Hitachi Modular PyD Autoanalyzer, Roche Laboratory Systems, Mannheim, Germany). Insulin was analyzed by chemiluminiscence using an automatic analyzer (DIAsource INS-IRMA; Nivelles, Belgium). Insulin resistance was calculated using the homeostasis model assessment (HOMA) index, as defined by the equation HOMA = fasting glucose (G0) (mM) x fasting insulin (I0) (μU/mL)/22.5. Adiponectin was analyzed in serum by ELISA using the HADK1-61K-A LINCOplex kit on a Luminex 200 System (Luminex Corporation, Austin, TX, USA).

Fatty acids were determined in the total lipids of both maternal and cord serum after Folch extraction (30). Gas chromatographic analysis was performed on a Hewlett-Packard, 6890 (Agilent Technologies, Inc. Palo Alto, CA, USA) equipped with a SP-2560 capillary column (100 × 0.25 mm id × 0.20 μm; Supelco, Sigma-Aldrich, St. Louis, MO, USA) and FID detection system. Helium was used as the carrier gas at a pressure of 290 kPa. Peaks were identified by comparison of retention times with appropriate FAME standards (Sigma-Aldrich, St. Louis, MO, USA).

Lysophospholipids (Lyso-PLs) were analyzed with flow-injection mass spectrometry (FIA-MS/MS). Ten microliter of serum and 10 μL of standard solution were diluted with methanol, containing internal standards for different lipid groups. After shaking and centrifugation, the supernatants were transferred and directly used for FIA-MS/MS analysis. Samples were analyzed with triple quadrupole mass spectrometer API4000 QTRAzLC/MS/MS System (Sciex, Darmstadt, Germany) coupled by an electrospray ionization source, which was used in both positive and negative mode, to a LC system (Agilent, Waldbronn, Germany). MS/MS analysis was run in Multiple Reaction Monitoring mode. The analytical process was post-processed by the Analyst 1.5.1 software.

### Protein Extracts for Western Blotting

Protein extracts were obtained from 30 μL of whole blood using 270 μL of cell lysis buffer, and from 15 μL of serum by adding also 285 μL of cell lysis buffer (Cell Signaling Technology, Danvers, MA, USA) containing 1 mM PMSF (Sigma-Aldrich, St. Louis, MO, USA). Samples were homogenized in a TissueLyser LT (QIAGEN, Barcelona, Spain) for 3 min and then centrifuged at 13,000 rpm, 4°C, for 15 min. As reference samples, the blood from three non-pregnant subjects, two women and a men, were also analyzed. Protein extracts from 100 mg of placental tissue were obtained as previously detailed (31). Protein was quantified by Bradford assay (32), and samples stored at −80°C until Western blotting analysis.

### Antibodies

Two primary rabbit polyclonal anti-MFSD2a antibodies were used (Abcam, Cambridge, UK, Ref.: ab105399 and Ref.: ab177881). We performed two incubations with the two different antibodies to corroborate that both of them were able to recognize the proper bands corresponding to MFSD2a protein. Anti-β-actin (Sigma-Aldrich, Saint Louis, MO, USA) and anti-GADPH antibodies (Abcam, Cambridge, UK, Ref.: ab8245) were also used. Secondary policlonal anti-rabbit antibodies conjugated with horseradish peroxidase were obtained from Santa Cruz Biotechnology (Santa Cruz Biotechnology, CA, USA).

### Western Blotting Analyses

Protein extracts (30 μg protein from placenta; and 8 μg from blood and serum) diluted in sample buffer, and protein standard (Dual Color Precision Plus Protein Standards, Biorad, Richmond, CA, USA) were resolved on 10% polyacrylamide gels, and transferred into polyvinylidene difluoride (PVDF) membranes (Millipore Corporation, Bedford, MA, USA), which were then blocked in PBS-T (phosphate saline buffer with 0.1% Tween-20) containing 2% BSA for 1 h at room temperature. Thereafter, membranes were incubated with anti-MFSD2A antibody (ab105399) 1:5,000 overnight at 4°C (33). Blots were washed with PBS-T and probed for 1 h at room temperature with the corresponding secondary antibody conjugated with horseradish peroxidase. Finally, membranes were stripped with Tris/HCL-Buffer pH 2.3 containing 0.1 M β-mercaptoethanol (Sigma-Aldrich, Saint Louis, MO, USA) and re-tested with antibodies to GADPH 1:1,000 for blood and β-actin 1:15,000 for placental samples, respectively, as loading controls. To quantify the level of MFSD2a in the samples, we run 12 samples in each gel, plus another sample as calibrator, respect to their loading control (23). In order to verify that the first primary antibody used (Abcam, Ref.: ab105399) properly recognized MFSD2a, we performed a second stripping of the membrane previously incubated with this anti-MFSD2a antibody and the anti-GADPH antibody, and then we performed another incubation with a different primary anti-MFSD2a antibody (Abcam, Ref.: ab177881). Data corroborated a similar band profile after using the two different anti-MFSD2a antibodies. Proteins were detected with a chemiluminescence kit following the manufacturer's instructions (Pierce ECL 2 Western blotting Substrate; Thermo Scientific, Rockford, Il, USA). Densitometry was performed on all blots to determine the density of the bands, using AmershamTM Imager 600RGB (GE Healthcare, Barcelona, Spain) and the Software: ImageQuant TL software version 8.1 (GE Healthcare, Barcelona, Spain). Relative protein level data were normalized against β-actin level in the case of placental MFSD2a, GADPH level for maternal blood MFSD2a, and albumin stained with Ponceau S solution (Sigma-Aldrich, St. Louis, MO, USA) in the case of serum.

### Statistical Analysis

The results are expressed as mean ± standard error of the mean (SEM). Data followed a normal distribution according to a Shapiro-Wilk test. Differences between control, GDM-Diet and GDM-Insulin groups were evaluated by ANOVA followed by *post-hoc* Bonferroni analyses. For the cesarean rate, a chi-square (*X*^2^) test was performed. The significance level was set at *p* < 0.05. Partial Pearson correlation analyses were also performed using as co-variables the sex and the BMI of children at birth. The statistical analyses were evaluated by the SPSS® 16.0 software package (SPSS, Chicago, IL). The minimum sample size to detect a significant difference between the groups with respect to the cord blood fatty acids (type I error α = 0.05 and type II error β = 0.2) was estimated to be 17 subjects/group, based on a minimal difference of 20% between means per the DHA in venous cord plasma (34).

## Results

### Maternal and Neonatal Anthropometric and Biochemical Parameters

Gestational age at delivery was significantly lower for both GDM groups compared with the controls due to the elective early pregnancy termination on these women attempting to avoid macrosomia. A tendency to higher cesarean rate in these patients was also observed (Control 26%, GDM-diet 30%, GDM–insulin 30%, *p* = 0.060). Determination of maternal biochemical parameters at recruitment (on the third trimester, before any treatment) showed that blood levels of glucose, insulin, TG and total fatty acids were significantly higher in GDM patients, especially in the GDM-Insulin group, than in the control group (Table 1) while maternal adiponectin was significantly lower in the diabetes groups (Table 1). Placental thickness, measured by ultrasound, and placental weight at delivery were also higher in both GDM groups, which might affect placental transport of nutrients (Table 1).

**Table 1:** Maternal and offspring characteristics and biochemical features.

**MOTHERS**				
	**Control (*****N*** **= 25)**	**GDM-diet (*****N*** **= 23)**	**GDM-insulin (*****N*** **= 20)**	***p***
Mother's age (years)	31.1 ± 0.9^b^	35.2 ± 0.8^a^	32.5 ± 0.8^ab^	**0.003**
Pregestational BMI (Kg/m^2^)	23.2 ± 0.8^b^	26.2 ± 1^ab^	28.2 ± 1.3^a^	**0.005**
BMI 3rd trimester (Kg/m^2^)	26.0 ± 0.7^a^	29.0 ± 1^ab^	30.6 ± 1^b^	**0.003**
Placental thickness 3rd trimester	35.9 ± 1.44	36.4 ± 1.74	39.7 ± 2.08	0.257
Placental thickness delivery	38.2 ± 2.1^a^	47.8 ± 2.4^b^	49 ± 2.4^bc^	**0.002**
Placental weight delivery (g)	582 ±24^b^	651 ± 26^ab^	674 ± 34^a^	**0.045**
Glucose 3rd trimester (mg/dL)	72.8 ±1.4^a^	80.6 ±1.8^ab^	84.0 ± 4.0^b^	**0.007**
Insulin 3rd trimester (mg/dL)	15.2 ± 1.4^a^	17.1 ± 1.7^a^	28.4 ± 5.0^b^	**0.004**
HOMA 3rd trimester	2.7 ± 0.2^a^	3.4 ± 0.4^ab^	5.8 ± 1.4^b^	**0.020**
TG 3rd trimester (mg/dL)	183 ± 17.7^a^	188 ± 10.6^ab^	240 ± 18.3^b^	**0.028**
Adiponectin 3rd trimester (mg/L)	8.31 ± 0.58^a^	6.34 ± 0.69^b^	6.14 ± 0.61^b^	**0.026**
Total FA 3rd trimester (mg/dL)	501 ± 19.4^a^	506 ± 17.3^a^	627 ± 44.5^b^	**0.003**
DHA 3rd trimester (%)	3.90 ± 0.16^ab^	4.02 ± 0.16^a^	3.32 ± 0.21^b^	**0.025**
Lyso-PL DHA 3rd trimester (%)	1.00 ± 0.06^b^	1.28 ± 0.11^a^	1.08 ± 0.06^ab^	**0.034**
**OFFSPRING**				
	***N*** **= 25 (12♂, 13♀)**	***N*** **= 23 (10♂, 13♀)**	***N*** **= 20 (14♂, 6♀)**	
Gestational age (weeks)	39.5 ± 0.15^a^	38.1 ± 0.3^b^	38.2 ± 0.2^b^	**< 0.001**
Z-score fetal AC 3rd trimester	−0.3 ± 0.2	0.6 ± 0.2	0.6 ± 0.2	0.075
Z-score fetal AC delivery	−0.3 ± 0.2	0.3 ± 0.2	0.4 ± 0.2	0.071
Z-score Birth weight	0.3 ± 0.2	0.4 ± 0.2	0.6 ± 0.2	0.482
Z-score Length baby	0.2 ± 0.2	0.7 ± 0.2	0.9 ± 0.2	0.099
Z-score BMI baby	0.1 ± 0.25	−0.3 ± 0.2	0.02 ± 0.2	0.474
Head circumference birth	34.8 ± 0.3	34.0 ± 0.3	35.0 ± 0.3	0.051
Head circumference 15 days	36.4 ± 0.2^a^	35.4 ± 0.3^b^	36.0 ± 0.2^ab^	**0.007**
Head circumference 1 month	37.9 ± 0.2	37.3 ± 0.3	37.5 ± 0.1	0.271
Head circumference 3 months	40.8 ± 0.25	40.0 ± 0.3	40.7 ± 0.2	0.083
Head circumference 6 months	43.7 ± 0.2	43.2 ± 0.25	43.65 ± 0.25	0.249
Head circumference 12 months	46.5 ± 0.2	46.0 ± 0.3	46.7 ± 0.35	0.219
Glucose cord (mg/dL)	69.4 ± 3.7	67.8 ± 3.5	76.3 ± 6.2	0.390
Insulin cord (UI/mL)	8.9 ± 1.7	11.3 ± 1.8	8.7 ± 1	0.453
HOMA cord	1.6 ± 0.5	1.6 ± 0.3	1.2 ± 0.2	0.706
TG cord (mg/dL)	42.1 ± 3.6^a^	32.1 ± 3.5^ab^	28.8 ± 2.2^b^	**0.015**
Adiponectin cord (mg/L)	28.99 ± 3.54	29.06 ± 3.81	24.80 ± 5.84	0.588
Total FA cord (mg/dL)	184 ± 9.4^a^	146 ± 4.5^b^	155 ± 5.7^b^	**0.001**
DHA cord (%)	6.51 ± 0.29^a^	5.70 ± 0.26^ab^	5.52 ± 0.26^b^	**0.029**
Lyso-PL DHA cord (%)	1.90 ± 0.09	1.74 ± 0.09	1.66 ± 0.08	0.152

*AC, Abdominal circumference; BMI, Body mass index; DHA, Docosahexaenoic acid; FA, Fatty acids; HOMA, Homeostasis model assessment; Lyso-PL, Lysophospholipids; TG, Triglycerides. Results are expressed as Mean ± SEM. ANOVA followed by Bonferroni test was used to assess differences between the groups. Different superscripts letters indicate significant differences between groups (p < 0.05) (boldface)*.

On the contrary, analyses of the cord blood showed that glucose levels were similar among the three groups, while TG and total fatty acids were both significantly lower in the two GDM groups, which was concomitant with an enhanced fetal adipose storage in these fetuses, as suggested by the higher fetal abdominal circumference (AC) observed by sonographic examination (Table 1). No significant differences were found in cord adiponectin among groups (Table 1). The percentage of DHA in cord blood was significantly lower in GDM-insulin offspring vs. control, whereas the proportion of DHA percentages in maternal serum at third trimester was higher in GDM-diet vs. GDM-insulin. DHA percentage in cord serum lyso-PL tended to lower values in GDM offspring (*p* = 0.152; Table 1), with a statistically significant difference in the direct comparison of GDM-diet vs. controls (*p* < 0.05), which suggests impaired placental transfer in GDM patients.

### Reduction of MFSD2A Protein Expression in Maternal Blood of GDM Patients

**Figure 1:**
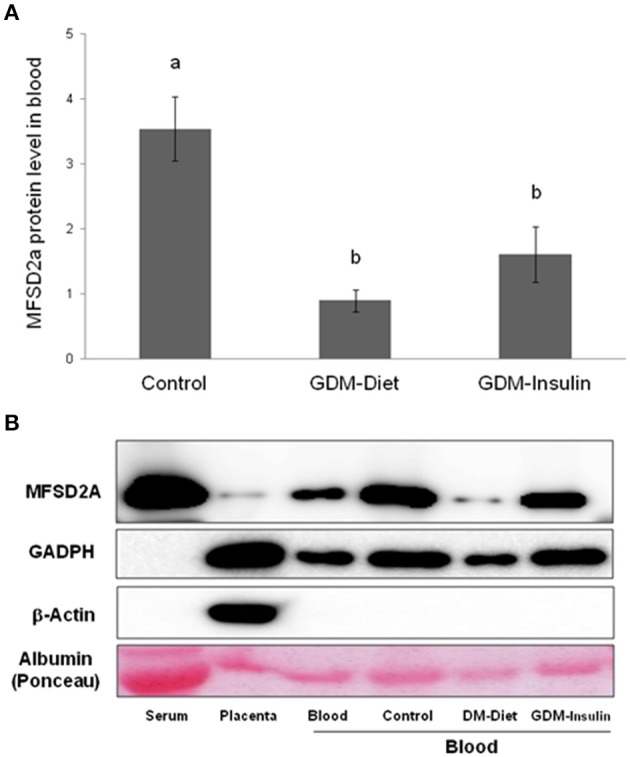
**(A)** Relative protein[[Inline Image]] level of MFSD2a in maternal blood of controls, GDM treated with diet and GDM treated with insulin (Normalized respect to GAPDH levels and calibrated respect to one referential blood sample). Results are expressed as Mean ± SEM. ANOVA followed by Bonferroni test was used to assess differences among the groups. Different superscripts letters on the bars indicate statistically significant differences (*p* < 0.05) among groups. *N* = 54 (24 controls, 15 GDM-Diet, and 15 GDM-insulin). **(B)** Example of Western blot analysis of MFSD2a, GADPH, β-actin, and albumin stained with Ponceau S solution expression in serum, placenta and blood from control pregnant women and GDM groups.

**Figure 2:**
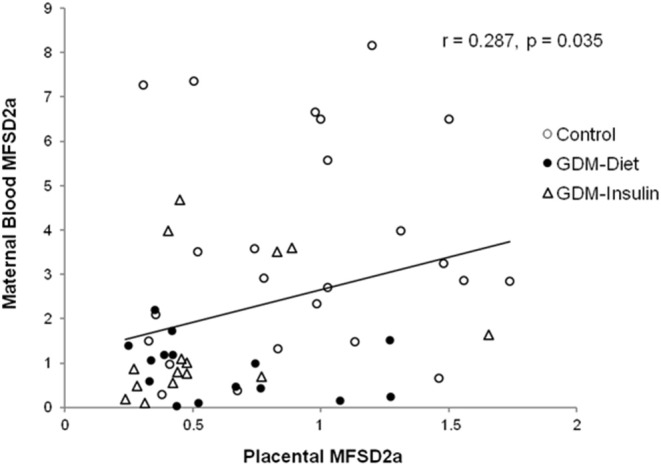
Correlation of the maternal blood MFSD2a protein level at the third trimester of pregnancy with the placental MFSD2a level at delivery. *N* = 54 placenta and maternal blood pairs (24 controls, 15 GDM-Diet, and 15 GDM-insulin).

The protein expression of MFSD2a was significantly lower in maternal blood from both GDM groups (Control 3.54 ± 0.50^a^, GDM-diet 0.89 ± 0.17^b^, GDM-insulin 1.60 ± 0.42^b^, *p* < 0.001; Figure 1A), which was in agreement with the significantly lower level of MFSD2a previously reported by our group in placental tissue obtained from the same cohort of GDM patients (Control 0.91 ± 0.08^a^, GDM-diet 0.56 ± 0.07^b^, GDM-insulin 0.49 ± 0.046^b^, *p* < 0.001) (23). Surprisingly, MFSD2a was also found in maternal serum of the three groups, but in this case no significant differences among groups were found (Control 1.07 ± 0.12, GDM-diet 1.73 ± 0.37, GDM-insulin 1.05 ± 0.17, *p* = 0.071; Figure 1B and Supplementary Material). Moreover, correlation analyses of the protein expression of MFSD2a in maternal blood samples and the corresponding placentas found a weak although statistically positive correlation (*r* = 0.287, *p* = 0.035; Figure 2), pointing toward the possibility of using maternal blood samples for the early detection of disturbances in placental MFSD2a expression during pregnancy.

### Association of MFSD2A Level in Maternal Blood With Neonatal Head Circumference, Biochemical Parameters and DHA Materno-Fetal Transfer

**Figure 3:**
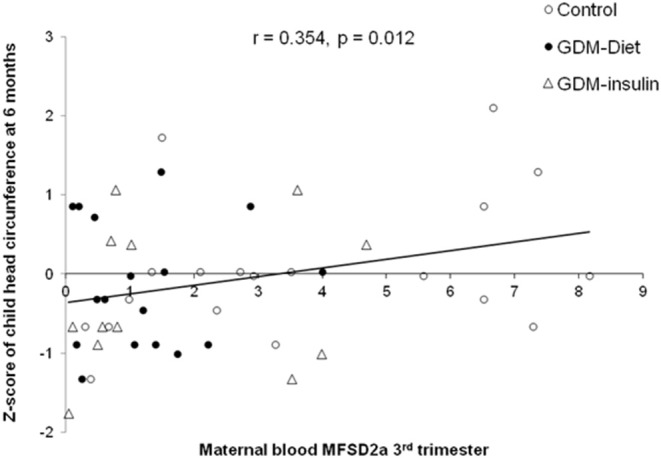
Correlation of the MFSD2a level in maternal blood at the third trimester with the child *Z*-score of head circumference at 6 months of life, adjusting by sex and BMI of children at birth. *N* = 48 maternal blood and head circumference pairs (19 controls, 17 GDM-Diet, and 12 GDM-insulin).

**Figure 4:**
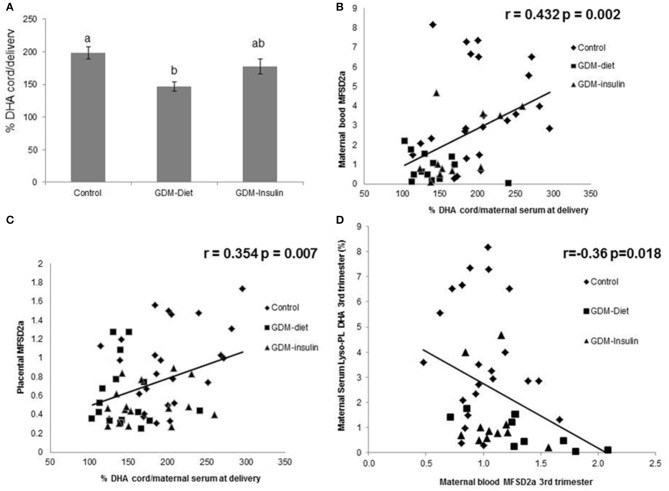
**(A)** Ratio cord/maternal serum DHA (%) in Control, GDM-Diet, and GDM-Insulin groups. Results are expressed as Mean ± SEM. ANOVA followed by Bonferroni test was used to assess differences among groups. Different superscripts letters on the bars indicate statistically significant differences (*p* < 0.05) among groups *N* = 63 (25 controls, 21 GDM-diet, 17 GDM-insulin) **(B)** Correlation of maternal blood MFSD2a protein level at the third trimester with ratio cord/maternal serum at delivery DHA. *N* = 48 pairs (24 controls, 13 GDM-Diet, and 11 GDM-insulin) (%). **(C)** Correlation of placenta MFSD2a protein level at the third trimester with ratio cord/maternal serum at delivery DHA (%) *N* = 57 pairs (25 controls, 16 GDM-Diet, and 16 GDM-insulin). **(D)** Correlation of blood MFSD2a protein level with DHA in maternal serum lyso-PL., = 42 pairs (22 controls, 9 GDM-Diet, and 11 GDM-insulin).

MFSD2a levels in maternal blood positively correlated to the Z-score of head circumference in children at 6 months of age (*r* = 0.354, *p* = 0.012; Figure 3) and also with head circumference at several time points during the first year of life (Table 2). Expression of MFSD2a in maternal blood tended to correlate also with several biochemical parameters measured in cord blood such as the level of proteins, TG, insulin, and HOMA (Table 2) but not with maternal adiponectin (*r* = 0.094, *p* = 0.530) or cord adiponectin (*r* = −0.009, *p* = 0.955). The ratio cord/maternal serum DHA (%) in total lipids at birth, which is an indicator of materno-fetal DHA transport, was lower in both GDM groups (Figure 4A) and was associated to MFSD2a expression in both maternal blood (*r* = 0.432, *p* = 0.002; Figure 4B) and placenta (*r* = 0.354, *p* = 0.007; Figure 4C). Furthermore, MFSD2a level of expression in maternal blood on the third trimester was inversely correlated to DHA in maternal serum lyso-PL (*r* = −0.360, *p* = 0.018; Figure 4D), which could reflect the activity of the carrier to uptake DHA from lyso-PL from the serum to the blood cells.

**Table 2:** Correlations of maternal blood MFSD2A protein levels with different child anthropometric and biochemical features during the first year of life.

	***r***	***p***
Head circumference at birth	−0.057	0.701
Head circumference at birth	0.137	0.197
Head circumference at 15 days	0.290	**0.033**
Head circumference at 1 month	0.308	**0.025**
Head circumference at 3 months	0.156	0.164
Head circumference at 6 months	0.335	**0.016**
Head circumference at 12 months	0.251	0.056
Total proteins cord	0.260	0.051
TG cord	0.279	**0.039**
Glucose cord	0.038	0.408
Insulin cord	0.320	**0.021**
HOMA cord	0.352	**0.012**

*HOMA, Homeostasis model assessment; TG, Triglycerides. Partial Pearson correlations adjusted by sex and BMI of children at birth are statistically significant at p < 0.05 (boldface)*.

## Discussion

In this study, we have demonstrated for the first time the expression of the MFSD2a protein in whole human blood and serum. Besides, we have detected a reduction of MFSD2a levels in maternal blood of GDM patients at the third trimester of pregnancy that correlates with a lower expression of MFSD2a in the placenta of these patients, which, in turn, would compromise DHA materno-fetal transport. In fact, the level of expression of MFSD2a in maternal blood during pregnancy is associated to the ratio cord/maternal serum DHA in total lipids at birth, which is a robust indicator of materno-fetal DHA transfer. In this regard, we have also found a reduction of materno-fetal DHA transport in GDM patients. Surprisingly, the level of MFSD2a in blood also correlated with the postnatal infant head circumference during the first 6 months of life, which could imply that alterations on MFSD2a levels during pregnancy might affect the normal development of the fetus and neonatal brain. Therefore, the determination of MFSD2a levels in maternal blood at the third trimester could be used as a potential biomarker for the early detection of disturbances in MFSD2a expression in pregnancy and the corresponding consequences upon the neurodevelopment of the child.

Quantitative analysis of MFSD2a in various human tissues have demonstrated its specific level of expression in several organs, showing 10-fold higher level of MFSD2a in placenta respect to many other tissues (22). However, the access to human tissues samples, such as placenta, during pregnancy for clinical analysis purposes is very limited. Therefore, more attainable biological samples, such as the blood, would represent a more suitable alternative for the study of MFSD2a and many other molecules that may be used as proteomic biomarkers for the clinical evaluation of gestational diabetes mellitus (35). In this regard, in the present study we have reported a lower level of MFSD2a protein expression in maternal blood of GDM patients (Figures 1A,B). This new piece of data obtained from the analyses of maternal blood is consistent with previous data obtained by our group that showed how MFSD2A level in placenta of these GDM subjects was reduced at delivery, affecting, in turn, DHA materno-fetal transfer (23). In addition, we have also found that MFSD2a expression in maternal blood at the third trimester correlates with MFSD2a expression in placenta. Thus, altogether, these data point toward the possibility of using the determination of MFSD2a level in maternal blood as an early indicator of pregnancy alterations and the posterior consequences caused by these alterations on the offspring.

MFSD2a protein has 12 transmembrane domains composed of two evolutionary duplicated six transmembrane units (36). We have detected by Western blotting assays different molecular weights bands corresponding to MFSD2a (~100, 70, and 50 KDa) that have been previously described in different cell types and tissues from human, mice, and pig, in which this protein showed different patterns of glycosylation that could imply different functions (18, 37–39); in our study the band analyzed in blood samples was of 100 KDa. Using three-dimensional structural models, it was suggested that MFSD2a undergoes conformational changes to pivot the translocation of lyso-PL from the outer leaflet to the inner leaflet of the membrane (36). Previously, our group determined that DHA percentage in the cord vein blood correlated with MFSD2a levels, suggesting a possible role in materno-fetal placental transfer of DHA (23, 40). In this study, in the third trimester, the expression of MFSD2a in maternal blood inversely correlated to lyso-PL DHA in maternal serum (*r* = −0.36, *p* = 0.018; Figure 4D), while it was positively correlated to the ratio cord/maternal serum DHA (%) in total lipids at birth, which is a solid indicator of materno-fetal DHA transport. These data suggest that the lower level of MFSD2a protein expression in blood cells of GDM patients would provoke a less efficient transport of lyso-PL DHA from the maternal serum to tissue cells such as blood cells, placental cells, brain cells or eye photoreceptors, among others. As reported recently by our group, lower placental MFSD2a protein expression was found in women with GDM treated either with diet or insulin. Even more, lower DHA levels in total lipids and in lyso-PL in cord blood were found also in the newborns from these GDM mothers, supporting the importance of lyso-PL and placental MFSD2a in the DHA availability to the fetus (23). Furthermore, the higher lyso-PL DHA percentage observed in the maternal serum of GDM-diet group support a potential disturbing maternal-fetal DHA transfer (23).

Infant head circumference is directly related to brain volume (41, 42). Some authors have previously reported the repercussion of MFSD2a mutations in various microcephaly syndromes, showing that the syndromes' severity correlates with the residual activity of the carrier, highlighting thus the key role of MFSD2a in the proper neurogenesis and maintenance of neurons, as the brain development and functionality is dependent on the Lyso-PL uptake (18–20). In this regard, homozygous mutation affecting a highly conserved MFSD2a residue associated with a progressive microcephaly syndrome is characterized by intellectual disability, spasticity, and absent speech (19). Individuals affected by this syndrome display significantly increased plasma concentrations of lyso-PL containing mono- and polyunsaturated fatty acyl chains, which is an indicative of their reduced brain uptake. In brain, lyso-PL may represent a physiological preferred carrier of DHA respect to non-esterified fatty acids (NEFA) (15, 43) and MFSD2a could be involved in such selective transport (15). Altogether, these findings indicate an essential role for lyso-PL in human brain development and function, and provide the first description of diseases associated with aberrant brain lyso-PL transport in humans (18). Moreover, Chan et al., have demonstrated in a mouse model that the adequate presence of MFSD2a at the blood-brain barrier (BBB) is required for normal postnatal brain growth and for maintaining plasma membrane phospholipid composition during brain development (44). Despite there were no changes in infant head circumference among groups, probably due to the low number of subjects to find differences in this clinical variable our current study shows how maternal blood MFSD2a level at third trimester correlate with postnatal infant head circumference of their offspring during the first 6 months of life (Figure 3 and Table 2) which is in agreement with Chan et al. (44). This result would suggest that the neurodevelopment of the child could be affected by the reduction of the MFSD2a level in the maternal blood during pregnancy. This association could also support a potential disturbed lyso-PL DHA transport to the brain, which could cause, in turn, alterations in the fetus neurodevelopment, but this point needs to be confirmed. In this regard, it would be very interesting to carry out further studies to evaluate the function of this carrier in blood cells and its role as a potential biomarker of the MFSD2a level in the brain. In addition, Harel et al., reported that MFSD2A-associated genotypes and phenotypes imply other factors like nutritional supplementation or modifying genetic factors, such as epigenetic modifications, which may influence the severity of the clinical presentation and proposed to investigate these compensatory mechanisms to treat microcephaly syndromes (20).

The correlation of maternal blood MFSD2a expression during pregnancy with several biochemical parameters in cord blood at delivery, such as cord proteins, TG, insulin, and HOMA (Table 2), seems to support that a proper expression level of MFSD2a improves the transport of nutrients across the placenta. These results are in agreement with the orphan transporter role of MFSD2a, whose ligands range from carbohydrates to aminoacids, but also different drugs, or organic anions, conferring it a great importance in metabolism regulation (38, 45).

MFSD2a carrier has been confirmed to be expressed in particular blood cells. Recently, Piccirillo et al. have reported that MFSD2a is expressed in T cells, being its expression essential for the correct functionality of CD8+ T Lymphocytes. These authors have observed that the expression of MFSD2a early during the CD8+ effector T cell immune response has critical long-term effects, as this transporter is required for the uptake of LPC into activated T cells, which is essential for the maintenance and turnover of memory CD8+ T cells (46). In this regard, a limitation of our study is that, although we have been able to analyze the expression of MFSD2a in whole maternal blood, we were not able to determine by flow cytometry or microscopy which populations of cells were actually those expressing this protein, and if the cellular profile expression of MFSD2a would be different in GDM patients and control subjects. In this sense, future studies will be necessary to clarify the level MFSD2a expression in each different blood cell population and their different metabolic status, which would bring further information about the nutrient transport across other tissues and its effect upon different cellular functions. Another limitation of this study is the size of the studied cohort, which may be increased in future studies to consolidate the results here presented.

Unexpectedly, we detected that MFSD2a was not only present in whole human blood, but also in serum, despite the fact that this transporter has been previously described exclusively as a membrane protein. To this point, it is uncertain whether this protein could be present in serum as part of the serum lipoproteins fraction or as part of other complex structures such as exosomes. In any case, MFSD2a levels observed in maternal serum did not correlate with the percentage of DHA in maternal plasma lyso-PL. In this matter, it is important to indicate that since MFSD2a protein levels were determined by Western blotting, the commonly used loading control proteins GADPH and β-actin were not detected in the serum samples. As this lack of loading controls was a limitation for the proper *serum* quantification of MFSD2a, we used Ponceau red staining of PVDF membranes to get the total loading of proteins as reference.

In conclusion, this study represents a first step to get a further insight in the relationship between the levels of MFSD2a in blood and other tissues. In addition, we found that levels of MFSD2a in maternal blood at the third trimester are disturbed in GDM patients, which may affect the proper transport of nutrients across the placenta and the cephalic perimeter of the offspring. So, the early detection of the MFSD2a level in blood during pregnancy related with placenta and DHA materno-fetal transport could be regarded as a potential and feasible biomarker of brain development before delivery. The analysis of this blood biomarker at the third trimester could be of great importance to detect problems in MFSD2a expression during pregnancy that would ultimately affect the offspring development, as well as this early data would be useful to select the best treatment options for the affected subjects.

## Data Availability Statement

The datasets generated for this study are available on request to the corresponding author.

## Ethics Statement

The studies involving human participants were reviewed and approved by Hospital Clínico Universitario Virgen de la Arrixaca Ethics Committee. The patients/participants provided their written informed consent to participate in this study.

## Author Contributions

EL and MS-C designed, conducted and evaluated the results. MS-S, MZ, MP-S, and JB-C were responsible for the collection of clinical samples and evaluation of the results. HD, BK, MR-P, MJR-P, AR-A and MS-C were responsible for the analysis and evaluation of the results. All the authors have participated in the writing of the manuscript.

### Conflict of Interest

The authors declare that the research was conducted in the absence of any commercial or financial relationships that could be construed as a potential conflict of interest.

## Supplementary Material

Supplementary Data Sheet 1 Representative examples of a Western blotting analysis of MFSD2a expression (upper image), and a mambrane stained with Ponceau S solution as loading control, in which the main band observed correspond to albumin content (lower image), performed with serum samples from healthy control pregnant women (C), and from GDM patients treated either with diet (D) or insulin (I).

## Abbreviations

AC: abdominal circumference
BMI: body mass index
DHA: 22:6n-3docosahexaenoic acid
FA: fatty acid
GADPH: glyceraldehyde-3-phosphate dehydrogenase
GDM: gestational diabetes mellitus
HOMA: homeostasis model assessment
LC-PUFA: long chain polyunsaturated fatty acids
MFSD2a: major facilitator superfamily domain containing 2A
lyso-PL: lyso-phospholipids
TG: triglycerides.
