# Supplementary figures and images for: Child Head Circumference and Placental MFSD2a Expression Are Associated to the Level of MFSD2a in Maternal Blood During Pregnancy
Source: Front Endocrinol (Lausanne). 2020 Feb 5;11:38. doi: 10.3389/fendo.2020.00038 (PMC7012934; doi:10.3389/fendo.2020.00038)

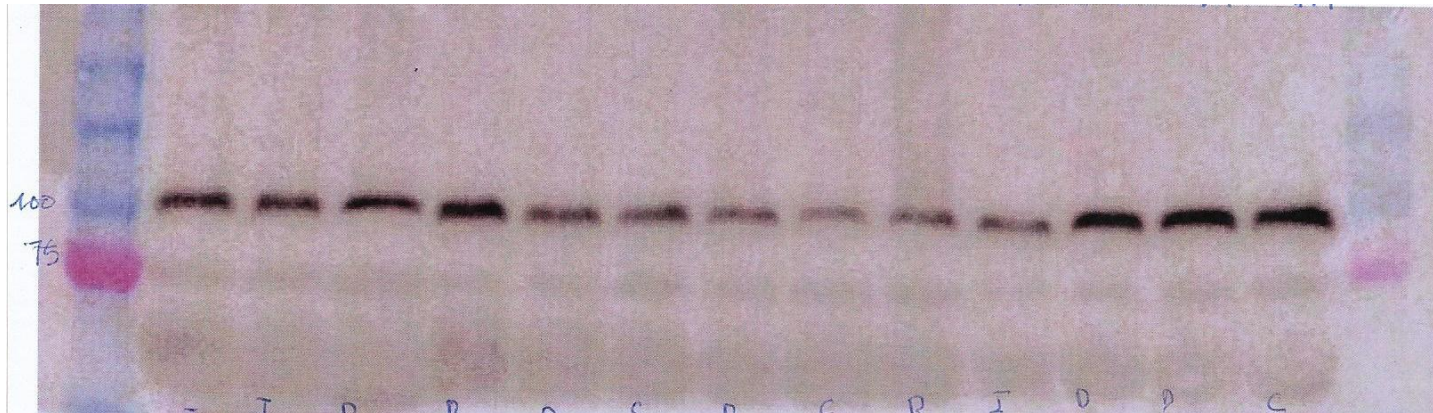

MFSD2a

C = Control

D = GDM-Diet

I = GDM-Insulin

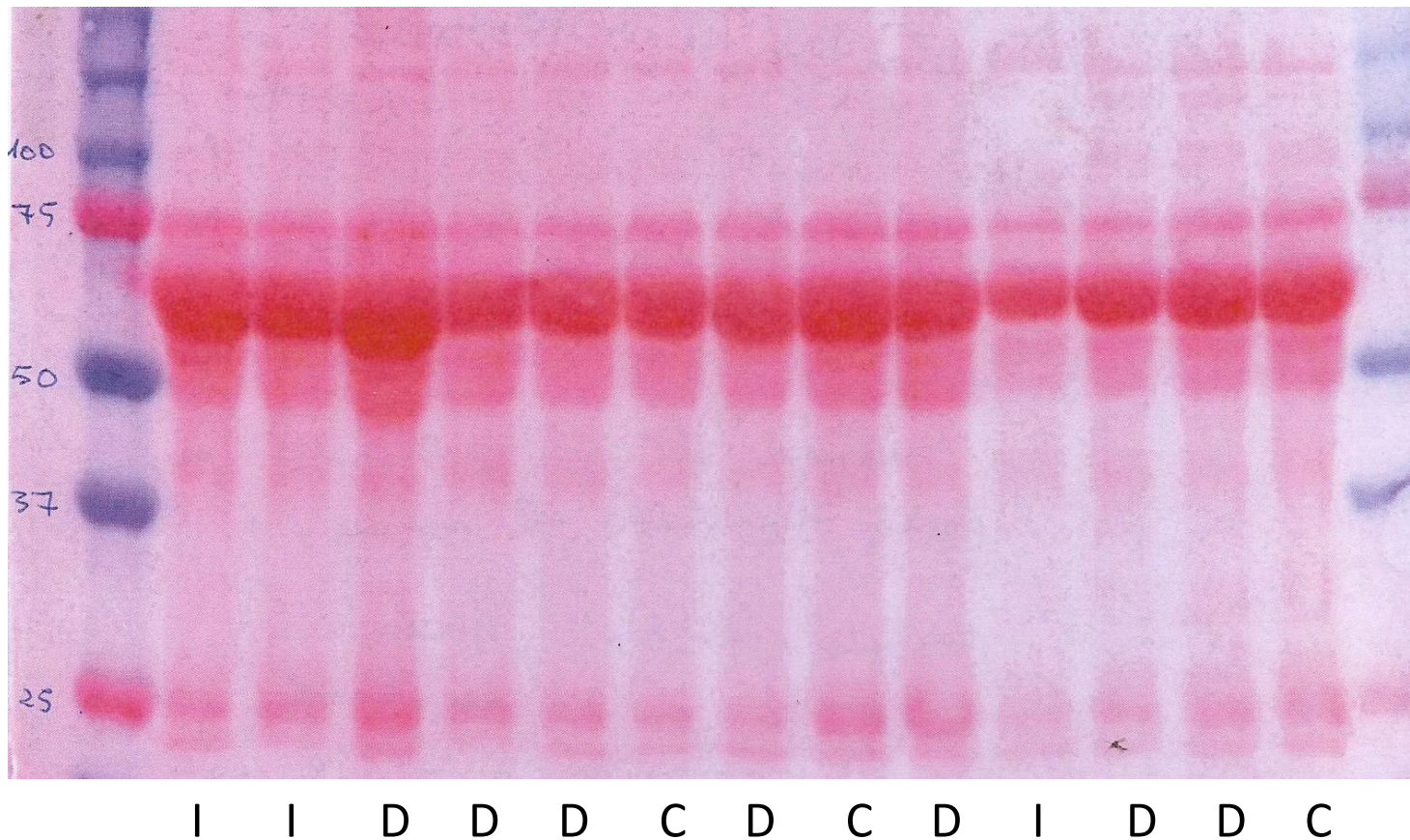

Albumin  
stained with  
Ponceau S  
Solution

Supplement: Supplementary Data Sheet 1 — Representative examples of a Western blotting analysis of MFSD2a expression (upper image), and a mambrane stained with Ponceau S solution as loading control, in which the main band observed correspond to albumin content (lower image), performed with serum samples from healthy control pregnant women (C), and from GDM patients treated either with diet (D) or insulin (I). [file Data_Sheet_1.PDF]
